# Interventions for reducing antimicrobial resistance in livestock in sub Saharan Africa: systematic review

**DOI:** 10.3389/fvets.2025.1702427

**Published:** 2025-12-17

**Authors:** Alice B. J. E. Jacobsen, Jane Ogden, Abel B. Ekiri

**Affiliations:** 1 Department of Comparative Biomedical Sciences, School of Veterinary Medicine, University of Surrey, Guildford, United Kingdom; 2 School of Psychology, University of Surrey, Guildford, United Kingdom

**Keywords:** Africa, livestock, antimicrobial resistance, intervention, antimicrobial stewardship

## Abstract

**Introduction:**

Although antimicrobial resistance (AMR) threatens the entire world, it disproportionately affects Low- and Middle-Income countries. The animal sector is a contributor to AMR and interventions for reducing AMR in this sector exist. However, in sub-Saharan Africa, there is limited information on AMR interventions targeting livestock.

**Methods:**

A systematic review was conducted following the PRISMA checklist to evaluate the existing evidence on AMR interventions, outcomes, motivators and barriers to adoption, and the strengths and weaknesses, with a focus on farmers and animal health professionals. The databases Web of Science, Scopus, and PubMed were searched. The articles were categorised into seven groups based on outcome measures: change in antimicrobial use (AMU) practices; change in AMR level; change in antimicrobial stewardship (AMS) practices; change in attitudes and perceptions concerning AMU, AMR, and AMS; change in knowledge concerning AMU, AMR, and AMS; change in surveillance strategies; and other.

**Results and discussion:**

A total of 546 articles were considered and, in the end, only five articles were eligible. The reported interventions focused on change in knowledge (3/5), change in AMS practices (2/5), surveillance (AMR and AMU) (2/5), and change in development of AMR (1/5). The motivators for adoption of interventions included social desirability and the barriers included lack of finance and lack of perceived sustainability of the interventions. The observed strengths of the reviewed studies included the use of One Health approaches, collaboration between researchers and the community, and involvement of a diverse study population. The observed weaknesses included self-reporting of outcome measures and lack of clarity in reporting. The financial impact and societal impact were not documented in any of the reported interventions. However, organisation culture was highlighted as having a positive impact on adoption of interventions in one study. The quality of the study designs was generally considered low.

**Conclusion:**

The findings revealed there is limited evidence on AMR interventions in the animal sector in Africa especially those focussed on change in AMU and change in development and/or spread of AMR. This gap suggests a need for well-designed and robust studies that assess and evaluate the impact of interventions and target animal health professionals and farmers in Africa.

## 1 Introduction

Although antimicrobial resistance (AMR) threatens the entire world, it disproportionately affects Low- and Middle-Income countries (LMICs). In 2019, Sub-Saharan Africa (SSA) had the highest rate of human deaths attributed to AMR (23.7 out of 100,000 deaths), about 50% higher than the global average (16.4 out of 100,000 deaths) (1). Unlike human, in animals, the burden due to AMR is not clearly documented but is currently being mapped by international organisations (2). It is estimated that by 2050 AMR could cause livestock losses large enough to equal the food needs of 746 million to 2 billion people (3). Inappropriate antimicrobial usage (AMU) in animals has been linked to AMR in humans and animals (4–6) around the world but is more prevalent in LMICs (7, 8).

Interventions aimed at tackling inappropriate AMU and the development and spread of AMR have been implemented in livestock with some success in both high-income and LMICs. For example, in Norway, Methicillin-resistant *Staphylococcus aureus* CC7 t091 was successfully eradicated on two pig farms (9). The interventions included total de- and repopulation of the pig herd and two protocols were compared. The first protocol was defined as high cost and high labour and involved total removal and renewal of internal surfaces of the floor area of the pig housing. The second protocol was defined as low cost and low labour and involved washing and disinfection of internal surfaces of the pig housing. Both protocols resulted in the eradication of livestock-associated methicillin-resistant *Staphylococcus aureus* on the pig farms (9). A further example is a 3-year intervention to reduce AMU in broiler production in Viet Nam (10). The intervention included provision of education to small-scale broiler farmers, use of antimicrobial replacement products, and flocks were given designated flock veterinarians. This intervention resulted in a reduction in AMU (66%) while decreasing mortality (40%) and increasing the body weight of the broilers (10). In SSA, however, there is limited evidence of related AMR interventions and the impact on the livestock sector.

Although interventions exist in select countries, several barriers limit the implementation and success of interventions to tackle AMR in the human and livestock sectors. At the global level, a lack of regulatory framework, funding, and human resources have been reported as barriers (8, 11). In SSA, barriers to the adoption of AMR interventions targeting healthcare professionals and animal health professionals have also been reported. A mixed-method study of pharmacists and physicians in a tertiary care hospital in Ethiopia assessed opportunities and barriers to implementing AMS and identified the following barriers: a lack of institutional and national policy, weak laboratory infrastructure and insufficient expertise in infectious disease specialists in the hospital (12). A cross-sectional study of veterinarians in Nigeria reported barriers to practices that promote AMS including failure to perform antimicrobial susceptibility testing and unavailability of laboratory services (13). Although these studies provide insight into perceived barriers to tackling AMR, little is known about barriers to implementation of structured interventions.

A systematic review was conducted to improve our understanding of the existing interventions aimed at tackling inappropriate AMU and the development and spread of AMR within the animal health sector in SSA, and the potential barriers to implementation. The targeted populations of interest for the AMR interventions were animal health professionals (veterinarians and para-veterinarians), farmers, and livestock (poultry, cattle, goats, sheep, swine, and aquaculture). A better understanding of the existing evidence, strengths and weaknesses of current interventions can help inform and guide the development of future interventions aimed at reducing AMU and improving AMS tailored to the local settings in SSA.

The specific study questions were:

1. What are the existing interventions aimed at tackling inappropriate AMU and the development and spread of AMR within the animal health sector in SSA and the related outcomes?
2. What are the motivators and barriers to the adoption of those interventions?
3. What is the documented impact of those interventions?
4. What are the strengths and weaknesses or limitations of those interventions?

## 2 Methods

**Figure 1:**
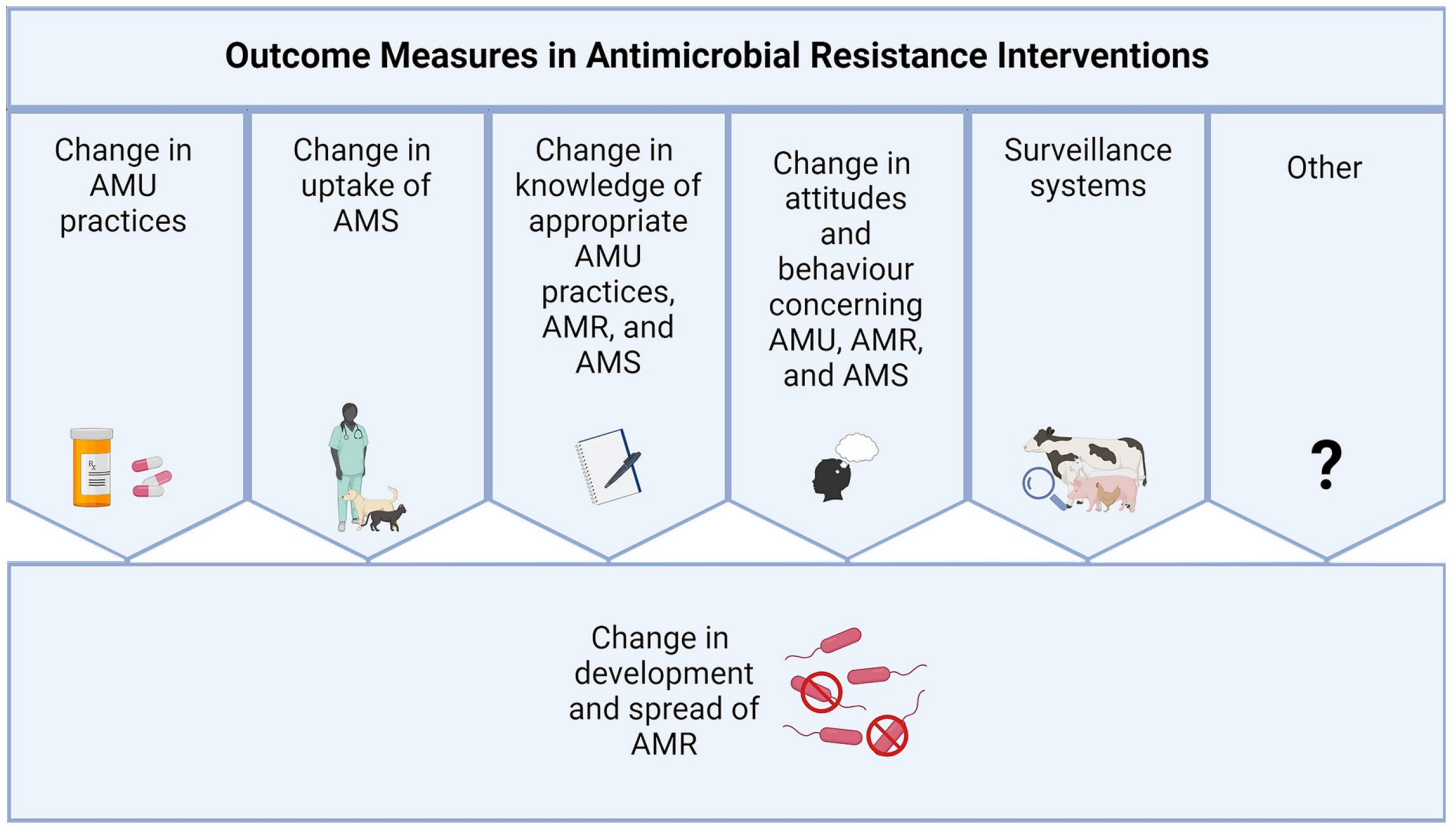
Seven outcome measures of interest for interventions on AMR, AMS and AMU.

A systematic review protocol was developed following the guidelines from the ‘Preferred Reporting Items for Systematic Reviews and Meta-Analyses Protocols’ (PRISMA-P)’ (14). For purposes of this review, to facilitate synthesis, the anticipated interventions from the reviewed studies were categorised into seven outcome measures: (a) change in AMU practices, (b) change in uptake of AMS, (c) change in development and spread of AMR, (d) change in knowledge of appropriate AMU practices, AMR, and AMS, (e) change in attitudes and behaviour concerning AMU, AMR, and AMS, (f) surveillance strategies, and (g) Other (Figure 1; Table 1) and as described in Jacobsen et al. (15).

**Table 1:** Outcomes for studies, and examples of interventions, included in systematic review.

Outcome	Examples
Change in AMU practices	Sanitation interventions, herd management plans, and educational interventions
Change in uptake of AMS	Changes in prescribing habits, adherence to AMS, and increased diagnostics
Change in development and/or spread of AMR	Clinical diagnosis, microbiological or resistance testing
Change in knowledge of appropriate AMU practices, AMR, and AMS	Educational intervention
Change in attitudes and perceptions to AMU, AMR, and AMS	Educational intervention
Surveillance systems	Surveillance of AMU or AMR
Other	Other relevant

### 2.1 Inclusion criteria

Primary research articles that investigated interventions to reduce AMR in the livestock sector in SSA and were published between 2002 and 2022 were included. Studies that targeted animal health professionals, farmers, or animals were included. One Health interventions were also included (i.e., animal health and environment and/or medical professionals). Interventions encompassing any of the seven focus areas described in Figure 1 and Table 1 were also included.

### 2.2 Exclusion criteria

Studies reporting interventions outside of the seven focus areas or that focused solely on human health and/or the environmental sector, were excluded. Reviews, commentaries, and editorials were excluded. Citations in relevant systematic review papers were checked for eligible references, but the reviews were excluded. Studies published before 2002 were excluded. Studies in any language other than English were also excluded.

### 2.3 Study objectives and population

The study objectives were structured following PICOS (population, intervention, comparison, outcome, study design) (16).

#### 2.3.1 Population

The study population included farmers, veterinarians, and para-veterinarians who were working with livestock (cattle, swine, aquaculture, and poultry). The interventions targeting animals may have been conducted in veterinary clinics or hospitals, or in farm settings. Both herd and individual-level interventions as well as regional and national interventions were included.

#### 2.3.2 Intervention

An intervention was included if the aim was to reduce AMR, increase AMS, reduce AMU, or change knowledge and/or attitude on AMR or AMS or AMU. The eligible interventions fulfilled the inclusion criteria and intervention focus as described in Supplementary Table 1 and in Jacobsen et al. (15).

#### 2.3.3 Comparison

There was no comparison in this study.

#### 2.3.4 Outcomes

For each of the four study questions, relevant outcomes were defined and assessed as described below.

For Question 1 (*What are the existing interventions aimed at tackling inappropriate AMU and the development and spread of AMR within the animal health sector in SSA and the related outcomes?*), the outcomes of interest were the defined primary outcome measures of the interventions reported in the studies (Table 1). These were sub-grouped further into secondary outcome measures (Supplementary Table 1; Figure 1).

For Question 2 (*What are the motivators and barriers to the adoption of those interventions?*), outcomes were grouped into motivators and barriers, and included factors such as personal circumstances, cost and/or finances, intrinsic factors, knowledge, health, and accessibility.

For Question 3 (*What is the documented impact of those interventions?*), four different outcomes were defined: socio-economic impact, cultural impact, societal impact, and negative effects. Socio-economic impact included whether the intervention had impact on social or economic factors of the target population. These include social standing, infrastructure, and economy (loss or increase of capital). Cultural impact pertained to whether the implemented interventions change habits, practices and beliefs within a given culture or group of people. Societal impact referred to impact of an intervention on people and communities (e.g., impact on education, healthcare, government services). Negative effects of the intervention were documented within a separate category. Impacts were only included if were documented within the original article.

For Question 4 (*What are the strengths and weaknesses or limitations of those interventions?*), outcomes of interest were weaknesses or limitations and strengths, and parameters assessed included design, implementation, sample size of target population and outcome measurements. Evaluations of strengths and weaknesses or limitations reported by the primary authors were also included.

### 2.4 Information sources

Database searches of PubMed, Scopus, and Web of Science were performed for relevant published literature. The bibliographies of included literature were also checked for relevant sources.

### 2.5 Search strategy

A combination of words relating to intervention, antibiotic, antimicrobial, bacteria, stewardship, farmer, veterinarian, para-veterinarian, and animal health professional along with the location of SSA was used to find appropriate literature. A description of the search strategy is provided in Supplementary Table 2.

### 2.6 Study screening

Titles and abstracts were screened within databases, following the exclusion and inclusion criteria, by one reviewer. Full-text articles were downloaded and managed in Mendeley. The same reviewer read the full text for eligibility and included or excluded the study accordingly. At the title and abstract and full text screening stages, a second reviewer screened a subset of the articles to reduce potential bias, with discrepancies discussed.

### 2.7 Data extraction

Data extraction was performed by one reviewer. A second reviewer corroborated this and performed extraction on a subset of articles (20%). The data extracted from the articles included: country of analysis, study setting (e.g., farm, hospital, region), authors, year of publication, type of publication, conflict of interest, type of study, the population of study (size, population type), length of study, and results relevant to study questions 1–4.

### 2.8 Data synthesis

Information from all reviewed studies was summarised qualitatively and descriptively and reported.

### 2.9 Strength of evidence and assessment of bias risk

The assessment of the risk of bias was performed using the Mixed Methods appraisal tool (17). The Mixed Methods appraisal tool covers the different study designs included in this review hence its selection. The tool includes two general questions and five questions for each study design that can be answered with ‘yes’, ‘no’, or ‘cannot tell. There is no score or cut off value (17). There are different questions for different studies designs, all denoted by different question numbers depending on the study design (1.1 to 5.5).

## 3 Results and discussion

**Figure 2:**
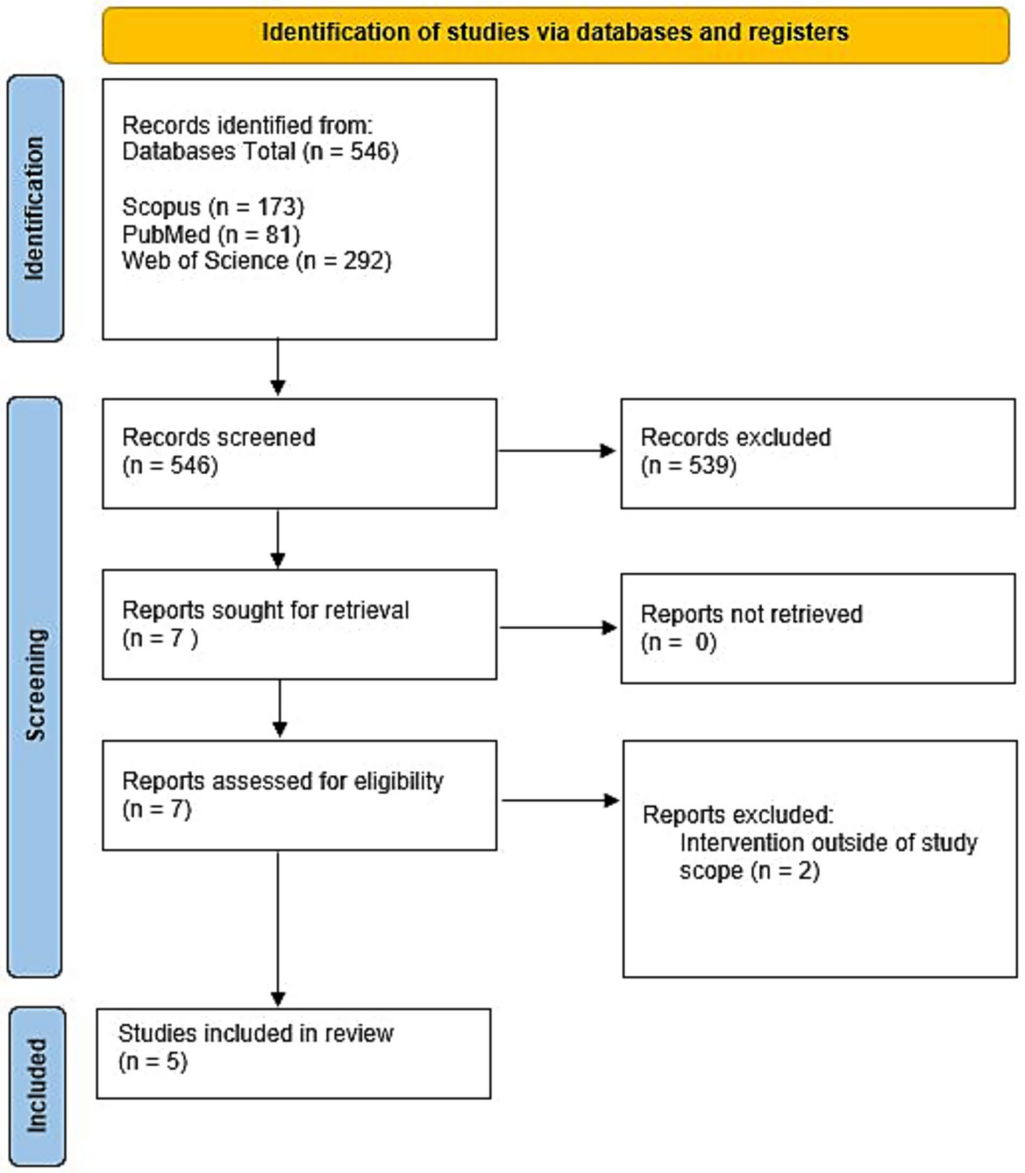
PRISMA SR flowchart.

A total of 546 studies were identified from databases. After title and abstract screening, 539 studies were excluded because did not satisfy the inclusion criteria. Seven articles satisfied the inclusion criteria, were retrieved, and eligibility was assessed further using full-text screening. Two studies were excluded at this stage because the reported interventions were outside the study scope. A citation search was performed for the five remaining articles, and no relevant articles were found. In the end, five studies were included in the review. The process of study selection is illustrated in a PRISMA flow chart (Figure 2).

The primary outcome measures were reported across the reviewed articles. Change in knowledge of appropriate AMU practices, AMR, and AMS (3/5) was the most frequently reported outcome measure, followed by the uptake of AMS (2/5), and surveillance systems (2/5). One intervention (1/5) reported reduced development and spread of AMR (Table 2). Secondary outcomes are also presented in Table 2, illustrating specifically the outcomes of the intervention.

**Table 2:** Primary and secondary outcomes by study.

Study	Primary outcomes measure	Secondary outcomes
Bangura et al. (22)	Surveillance strategies.	Surveillance of AMU/AM sales
Chinwe et al. (21)	Change in development and spread of AMR.	Reduced frequency of resistance genes within bacterial strains.
Frumence et al. (20)	Change in knowledge of appropriate AMU practices, AMR, and AMS.	Change in knowledge of appropriate antimicrobial use practices. Change in knowledge of AMR.
	Surveillance strategies	Surveillance of AMR.
Musoke et al. (18)	Change in uptake of AMS.	Change in prescribing habits. Increased adherence to guidelines. Increase in frequency of use of diagnostics, e.g., sensitivity testing. Other (sanitation).
	Change in knowledge of appropriate AMU practices, AMR, and AMS.	Change in knowledge of appropriate antimicrobial use practices. Change in knowledge of antimicrobial stewardship. Other (dosage of antibiotics).
Roulette et al. (19)	Change in uptake of AMS.	Other (dosage of antibiotics).
	Change in knowledge of appropriate AMU practices, AMR, and AMS.	Other (dosage of antibiotics).

Two interventional studies were conducted on a national level, and three studies were conducted on a small scale (i.e., herd level or small group of the target population) (Table 3). The interventions were conducted in Tanzania (2/5), Nigeria (1/5), Uganda (1/5), and Sierra Leone (1/5) (Table 3). Two studies were designed as before and after studies with no control group (non-controlled before and after studies) and the remainder were a descriptive study (1/5), a case–control study (1/5), and a qualitative study (1/5).

**Table 3:** Country, level of scale of intervention and targeted populations of the reviewed studies.

Study	Country	Scale	Study design	Targeted population	Other populations
Bangura et al. (22)	Sierra Leone	National	Descriptive	Livestock (multiple species^a^)	N/A
Chinwe et al. (21)	Nigeria	Small Scale	Case–Control Study	Livestock (broiler birds)	N/A
Frumence et al. (20)	Tanzania	National	Qualitative	Animal health professionals (Private practice, Veterinary Council of Tanzania)	Other related key players (ministries, laboratories, government agencies, international organisations, professional councils, health facility representatives)
Musoke et al. (18)	Uganda	Small Scale	Non-controlled before and after	Animal health professionals (veterinarians and para-veterinarians)	Medical health professionals, community health workers, students
Roulette et al. (19)	Tanzania	Small Scale.	Non-controlled before and after	Cattle farmers (men)	Milk pasteurisers (women)

^a^Cattle, dog, donkey, fowl, goat, goat and sheep, horse, pig, rabbit, sheep.

The targeted population in the studies was animal health professionals (2/5), livestock (2/5) and farmers (1/5) (Table 3). Most of the studies included populations outside of the target population of the review. The additional populations included medical health professionals, ministerial workers, and students.

With only five studies being found eligible, this review indicates few AMR interventions have been implemented in the animal sector in SSA. The reviewed interventions were also mainly on a smaller scale, and few (2/5) studies were conducted on a national level indicating a narrow reach of the studies. Despite the few reported interventions on AMR in SSA, the inclusion of different study populations, the use of a One Health approach, and conduct of national-level interventions were positive attributes of the reported AMR interventions. Additional well-designed intervention studies addressing understudied outcome measures such as change in AMU, AMS and development and spread of AMR in the livestock sector, are needed.

### 3.1 What are the existing interventions and the related outcomes?

Overall, there was an overlap in the focus of interventions and outcomes in three of the five reviewed studies. The details of the reported interventions and the outcomes are described below, and the primary and secondary outcome measurements are summarised in Table 2.

In the first study, training in the form of workshops about AMR and sanitation was provided to 76 medical health professionals and 10 animal health professionals in the Wakiso district of Uganda (18). The trainings were delivered as case studies and group discussions. No comparison group was used, but a follow-up evaluation of 80 medical and animal health professionals was conducted. The timeframe for this post-evaluation was not reported. The outcomes included a 16.1% increase in health professionals who could correctly define AMR and a 52.1% increase in participants who were aware that non-compliance with hand hygiene is a contributing factor to AMR. In a further evaluation of 68 health professionals, 3 months after training, change in the following practices were reported: improved handwashing (57.3%), increased use of guidelines for antimicrobial prescribing (52.9%), higher frequency of samples sent to the laboratory (35.3%), and reduction in antimicrobial drugs given per patient (51.5%).

The second study reported increased knowledge of AMR and change in uptake of AMS in 102 women and 60 male farmers from Maasai communities in Tanzania (19). No comparison group was used. The intervention was provision of information on AMR to men and women. In addition, men received dose charts for antimicrobials and tape measures to estimate livestock weight, while women received thermometers to determine temperature for milk pasteurization. Both groups were trained on how to use their intervention and given protocols to follow. The outcome was measured at a two-month follow-up. Women had retained 14% of the knowledge around AMR that they were given at the beginning of the intervention while men had retained 30% of the information. However, when asked to demonstrate the use of thermometers, the proportion of women that used thermometers correctly (70%) was more than men that performed dosage steps correctly (18%) (19).

The third study assessed the implementation of a national action plan on antimicrobial resistance in Tanzania (20). In-depth interviews were carried out with 111 key stakeholders including animal health professionals from both government and private practices. Other stakeholders interviewed were policymakers, regulatory authorities, laboratory authorities as well as on-the-ground workers (i.e., livestock officers, pharmaceutical technicians, and health centre workers). The interview included discussions around knowledge change and surveillance strategies for AMR. Other aspects discussed included AMS guidelines in the human sector, education of medical professionals on AMU and AMR, and the creation of public awareness around AMR (20). No comparison group or follow-up was conducted. Findings concluded that within the animal sector, surveillance sites for AMR had been developed and guidelines for AMU had been created.

The fourth study examined practices to reduce AMR in 100 broiler birds on a poultry farm in Nigeria (21). The intervention involved provision of feed with antibiotic additives to broiler birds (*n* = 100) and assessing the level of *E. coli* resistance compared to the control group (*n* = 100) that was given feed without antibiotic additives. Cloacal swabs were taken from broiler birds on day 20 and 40 to test for *E. coli*. On day 20, 14% of birds fed antibiotic additives had *E. coli* in feacal samples whereas the control group had a lower percentage of *E. coli* in feacal samples (8.5%). On day 40, carriage of *E. coli* was reported in 19% of the birds fed antibiotic additives compared to 4% in the control group (21).

The fifth study reported a surveillance strategy that involved recording data on AMU in the livestock population of Sierra Leone across 15 districts (22). The intervention included preparing and submission of weekly reports of AMU and livestock disease by community animal health workers over a duration of 35 weeks. The weekly reports were submitted through the district veterinary officer and livestock assistants to the Animal Health Epidemiology Unit of the Ministry of Agriculture and Forestry. Of the expected 525 weekly reports over the study period, 88% of the reports were available in 2020, up from 1% in 2016–2017 (22).

The outcomes linked to the reported interventions varied across the five studies. In three studies, the primary outcome was ‘change in knowledge surrounding AMR, AMU and AMS’. The reason for focus on the outcome ‘change in knowledge surrounding AMR, AMU, and AMS’ in the three studies may be due to this type of interventions being less expensive and resource-heavy to implement compared to other interventions (e.g., interventions focused on change in spread or development of AMR or change in AMU practices). There were no interventions in this review that focused on change in AMU practices. Understanding change in AMU practices is important because it can reveal if a given intervention is effective in driving reduction in AMU, and if not, such information can guide revisions in existing interventions or the development of new interventions. Similarly, understanding if there is change in spread or development of AMR could inform if an intervention reduces the burden of AMR. Future intervention studies can consider assessing change in AMU practices and change in the development and spread of AMR to provide data on relevant indicators of change in risk of AMR.

### 3.2 What are the motivators and barriers to the adoption of interventions?

A range of motivators and barriers to the adoption of AMR interventions were reported by the reviewed studies (Table 4). The reported motivators were broadly categorised by the investigators of the current study into social desirability (2/5), accessibility (1/5), knowledge (1/5), personal circumstances (1/5), and finance (1/5), while the barriers were categorised into accessibility (3/5), finance (3/5), sustainability (3/5), and knowledge (1/5).

**Table 4:** Motivators and barriers to the adoption of the interventions in reviewed studies.

Study	Motivators	Barriers
Bangura et al. (22)	Social desirability/responsibility (Government could assess whether AMU is in line with AWaRE.)	Finance (Required national network of paid workers to submit weekly reports.)
Chinwe et al. (21)	*Not enough information provided in article about motivators*	Knowledge (AM not illustrated on the bags of feed.)
Frumence et al. (20)	Social desirability/responsibility (Government was able to submit information to global registers and meet targets for national action plans signed.)	Finance and Accessibility (Inadequate resources.) Sustainability (Funded externally) Accessibility (Hard to breach gaps between sectors.)
Musoke et al. (18)	Accessibility (Short time spent (2 days) knowledge gained.) Knowledge (Discussion and knowledge across professions.)	Finance and Accessibility (cost for travel, time out of work, logistics of gathering). Sustainability (Affected by the involvement of external collaborators.)
Roulette et al. (19)	Personal circumstances (More children resulted in increased use of thermometers.) Finance (Wealth associated with increased retention of knowledge.)	Accessibility (High illiteracy limited use of dosage chart.) Sustainability (Affected by the involvement of external collaborators.)

#### 3.2.1 Motivators

Social desirability/responsibility was identified as a possible motivator for the implementation of interventions at a national level. Specific examples of interventions were a national action plan tackling AMR and a surveillance strategy focused on AMU (20, 22). Social responsibility is the desire to want to present in a more favourable, socially accepted fashion (23). A study that investigated the RESET intervention model in Dutch dairy farmers reported that social pressure, ‘doing the right thing’ and ‘being perceived as doing the right thing’, was a strong motivator for intervention success (24). Social responsibility may also influence the implementation of AMR interventions at the national and global levels. Out of the 195 countries in the world, 135 countries have created an AMR national action plan (NAP) (25). The NAPs provide guidance to governments to lay out their priorities and actions to tackling AMR (26). In addition, 103 countries have enrolled in the Global Antimicrobial Resistance and Use Surveillance System (GLASS). GLASS is a surveillance system for AMR and AMU (27). All participating countries submit AMR and/or AMU data to GLASS. GLASS uses the AWaRe (Access, Watch, Reserve) system to classify antimicrobials (28), with the aim being 70% of AMU from the Access class by 2030 (29). In 2022, 59% (*n* = 39) of countries reporting to GLASS used 60% or more antimicrobials from the Access class (27). Considering the perceived benefit of social responsibility, the increasing number of nations with NAPs s and/or joining GLASS may motivate other nations to do the same. More recently, the Quadripartite, the World Health Organization, World Organisation for Animal Health, and The United Nations Food and Agricultural Organization and The United Nations Environment Programme, have signed the One Health Joint Plan of Action, running from 2022 to 2026, ensuring collaboration and accountability addressing AMR across the sectors.

Accessibility was classified as a motivator for the target population to participate in educational workshops and discussions in the study by Musoke et al. (18). This intervention required an intense but short time frame in which the target population was required to be present to participate in the educational workshops and discussions. This meant that participants only had to give up a short time frame to gain knowledge and participate in the intervention. Ensuring accessibility to an intervention can help facilitate the uptake of an intervention.

The gaining of knowledge through discussion and knowledge sharing across professions was identified as a motivator in a One Health educational intervention in Uganda (18). However, it is recognised that education and knowledge alone are not enough to change behaviour. In Roulette et al. (19), a link between wealth/finance and knowledge retention was observed and hypothesised to be due to a higher likelihood of direct or indirect access to education and therefore may not be a motivator (19). In a review of 43 articles, that explored AMU interventions in human medicine in LMICs, knowledge and education were the most targeted intervention activities (30). Lack of education is perceived by medical health professionals in both LMICs and HICs as the biggest barrier to AMR reduction (31). However, in other public health campaigns such as smoking, education was deemed not enough and the reduction in smoking was primarily attributed to taxation (32). Extrapolating the above learnings to AMR in the livestock sector, education, even though perceived as important by those prescribing antimicrobial drugs, may not translate into change in behaviour considered necessary for the reduction of AMR. This finding suggests a need to include more than education in an intervention to increase the likelihood of achieving the intended change.

Personal circumstances were identified as a motivator. In Roulette et al. (19), having more children was reported as a motivator to use a thermometer to ensure correct pasteurisation of milk. The authors presumed this was due to a desire to maintain child health (19). Child health was reported as a motivator for participation in other public health interventions. In a qualitative study of motivators and barriers to creation of smoke-free environments, a growing understanding of the risk of second-hand smoke to children and the concern that children may become smokers were perceived as enablers to creating smoke-free environments (33). Similarly, emphasising the human health benefits of an AMR intervention with respect to human health and specifically child health may facilitate appreciation of the importance of AMR interventions.

#### 3.2.2 Barriers

Lack of accessibility was reported as a barrier to adopting interventions in three studies (Table 5). The reported barriers under accessibility were inadequate resources, and illiteracy amongst the target population. Despite differences across animal and human sectors, the challenges and barriers to AMR interventions are common. A systematic review of nine pharmacist-led AMS interventions in SSA (34) reported similar challenges to those reported in the animal sector shown in Table 5. A lack of laboratory and technology infrastructure, documentation of patient treatments, as well as lack of time and resources were identified as barriers to pharmacist-led AMS interventions (34). This study illustrates that some barriers (e.g., lack of resources) are common across target populations in both human and animal health care. The findings illustrate the importance of considering the capacity of a target population to implement a given intervention (such as do they have the required resources?, are they literate to undertake an educational intervention?) as well as addressing common gaps between sectors, where appropriate.

**Table 5:** Strengths and weaknesses or limitations by study.

Study	Strength	Weakness
Bangura et al. (22)	National study over 7 months period. Evaluation of surveillance.	Unaware of margin of error for reporting (unclear what is not being reported). No uniform terminology. Unclear if all community animal health workers are in reports. No information on how much researchers interfered/biased reporting. Self-reporting.
Chinwe et al. (21)	Implemented in field conditions within LMIC.	Unclear wording around methods and percent of carriage of *E.coli.* Short time frame, no information on animal health and production.
Frumence et al. (20)	One Health focus. Unique review of NAP in SSA. Large pool of interviews with diverse backgrounds.	No environmental stakeholders were involved in NAP. Donor-funded programme results in a limited timeframe and ability to continue intervention post funding-period. Limited infrastructure to implement NAP into.
Musoke et al. (18)	One Health focus. Part of an existing project (10-year collaboration). Reporting of positive effects on organisational culture.	Low participation of animal health professionals compared to medical health professionals. Improvements were self-reported and so were potentially biased. Unclear if follow-up includes animal health professionals.
Roulette et al. (19)	Assessed whether knowledge retention translated into action. The intervention was part of an existing project which promotes sustainability.	Shorter period (2 months). Follow-up was performed in dry season when there is less use for women’s intervention (thermometer). Self-reporting of use.

An additional barrier was related to the context in which animal health professionals and animal patients interact as part of veterinary service delivery. The delivery of health services differs between the animal and human sectors. Animal health professionals and animal patients (specifically livestock) are typically spread across large distances, and the health provider often travels to the respective locations. Whereas within a hospital, human patients usually come to one central place (hospital) to receive medical care, which facilitates the ability to distribute information and guidelines, and provides a conducive environment to discuss clinical decisions. In both HICs and LMICs, the ability to communicate and interact with colleagues is an integral part of health interventions (34–36), and this is easier in human hospital settings compared to livestock farm settings. Alternative ways to encourage engagement among animal health professionals in relation to communication of messages on AMR may help support uptake of interventions.

A lack of ongoing financial support and a lack of sustainability were reported as barriers to adopting interventions in three studies. In Bangura et al. (22), the financial capacity to pay a national network of workers to report results was identified as a barrier (22). Whereas in Musoke et al. (18), finance was considered a barrier due to the cost of travel and time off work for participants to attend workshops (18). The intervention by Frumence et al. (20) was funded externally with time-limited funding (20). Lack of funding is a common barrier both to participants of interventions (e.g., travel costs, time off work, inadequate resources) and to implementers of an intervention. If interventions are not perceived by participants as worthwhile or feasible due to lack of or limited funding, their desire to invest in and participate in the intervention may be reduced. Careful consideration of required funding and timeline of an intervention should therefore be considered at the design stage to maximise the likely commitment of participants and chances of successful implementation.

Sustainability of interventions is also an important consideration. In two of the studies, Musoke et al. (18) and Roulette et al. (19), the interventions were developed with external collaborators (18, 19). It is plausible that continuation plans for the interventions were in place for interventions included in this review. However, it is not documented. Clear commitment from all involved locally and externally and making long-term plans and communicating these to people involved may help increase sustainability of implemented interventions.

The intervention reported by Frumence et al. (20) was funded externally (20). Inadequate funding or funding that wanes after the end of the intervention can put sustainability of interventions at risk because without funding an intervention may not continue and therefore may not be sustainable (18–20). External funding is often needed to implement interventions in LMICs. However, the benefits and risks of using external funding in relation to sustainability should be weighed up carefully before implementing an intervention.

Knowledge, or lack of information related to antibiotics in feed was reported by Chinwe et al. (21). The authors reported that poultry feed bags lacked information on whether the feed contains antibiotics. This meant that farmers could not tell if the purchased feed contained antibiotics unless the feed was tested for antibiotics in a laboratory.

### 3.3 What is the documented impact of the interventions?

The impacts documented within the reviewed studies were described in the present study as socio-economic impact (19), cultural impact (18), and negative effect (20). Impact was not documented in the other two studies (21, 22).

#### 3.3.1 Socio-economic impact

Roulette et al. (19) found that knowledge retention within the intervention was associated with wealth, a potential socioeconomic impact (19). However, as the wealth was presumably present before the intervention, it is unlikely to be a result of the intervention. The study postulates that having access to education before the intervention is the true influencing factor, rather than wealth (19). Identifying if wealth is the true catalyst to knowledge retention, or if this is a confounding factor, may help identify factors that increase retention of knowledge of interventions.

None of the reviewed studies investigated the direct financial impact of their implementation. Financial impact is an important factor to consider because it can affect the sustainability of an intervention. Along with other factors (such as rules and regulations, education, information, social pressure, and tools), finance plays an important role in incentivising participation in interventions (24). The financial impact of an intervention is therefore an important factor to consider in the evaluation of interventions.

#### 3.3.2 Cultural impact

No studies reported having a cultural impact. However, Musoke et al. (18) indicated that participants expressed that change in organisational culture had occurred through the intervention, adopting new practices on AMS in line with national guidelines (18). Other studies have researched organisational culture in this context. Guo et al. (37) and Ukawa et al. (38) studied organisational culture and AMU in the human sector in two high income countries, Singapore and Japan, respectively, (37, 38). Guo et al. (37) investigated AMU decision-making of doctors in primary care and reported that a change in organisational culture helped to create and sustain personal values (37). Personal values were perceived as the most important factor influencing antimicrobial prescribing choices (37). Ukawa et al. (38) found that acute care hospitals with high scores in organisational culture were more likely to follow Japanese and Centers for Disease Control guidelines around perioperative AMU (38). The positive impact of change in organisational culture seemingly creates a more sustainable change than if singular individuals within organisations change practices.

#### 3.3.3 Societal impact

No societal impact was documented in the reviewed studies. Musoke et al. (18) included the public (school pupils and university students) as part of their target population but did not report the impact of the intervention on this group (18).

#### 3.3.4 Negative effects

No negative impacts were documented in the reviewed studies. However, although not directly a negative impact, multiple key opinion players in the study by Frumence et al. (20) expressed concerns about the intervention being donor-funded rather than government-funded (20). Interviewees reported an insecurity about what will happen after the funding runs out (20). As discussed previously, uncertainty around funding, may result in key stakeholders being less likely to engage with the intervention as may feel their time and effort may go to waste or that the intervention is not sustainable.

### 3.4 What are the strengths and weaknesses or limitations of the interventions?

The studies revealed strengths or weaknesses summarised in Table 5 and discussed in the subsections below.

#### 3.4.1 Strengths

The application of a One Health approach was observed in two of the reviewed interventions and was considered a strength. The One Health High-Level Expert Panel defines One Health as “*an integrated, unifying approach that aims to sustainably balance and optimize the health of people, animals and ecosystems*” (39). In the intervention reported by Frumence et al. (20), multiple sectors were reportedly involved in Tanzania’s National Action Plan for AMR. These multiple sectors included public health, livestock, and fisheries. In the educational intervention focussed on AMS reported by, Musoke et al. (18) the following key players from different sectors were included: animal and medical professionals, community workers, and the public (students) (18). However, the environmental sector was not included (18). AMR is a challenge across humans, animals and the environment. The application of a One Health approach in the development and implementation of AMR interventions can therefore help align efforts across the relevant multiple sectors.

Collaboration is a strength that appeared to contribute to the successful implementation of interventions, based on the reviewed studies. Two of the reviewed interventions were part of an ongoing collaboration (18, 19). Collaboration allows for sharing of knowledge, upscaling of skills by and across collaborators, and pooling of resources to maximise efficiency and to increase capacity across the participating partners. Although benefits exist, collaborations between local and external partners can be associated with risks as well. For example, the knowledge gained through the intervention may not be utilised within the community where the intervention took place but rather end up being used elsewhere far from that community or may end up only benefiting the external collaborator.

The use of qualitative study methodology in the design and implementation of interventions was considered a strength. Qualitative studies gather a diverse understanding of intervention outcomes and impacts and may offer insight into a broad range of aspects, including participants’ understanding of AMR, and their thoughts on motivators and barriers. The study by Frumence et al. (20) was a qualitative study that performed 111 interviews (20). The interviews were diverse across sectors but also vertically within sectors. Interviews included a wide range of participants at the ministerial level involved in creating and implementing policy, to laboratory technicians, livestock officers, dispensers, and those working on policy at the community level. This allows for cyclical feedback from those at the community level to those creating and making decisions at the higher levels which promotes an understanding of how a given policy related to an intervention works on the ground or at the community level. This is important when considering how to improve existing interventions and how to implement new ones.

#### 3.4.2 Weaknesses

The weaknesses observed in the reviewed studies are summarised in Table 5. Self-reporting of outcome measures was noted in two studies (18, 19). In Musoke et al. (18), participants self-reported change in the use of AMS (e.g., sending samples to the laboratory before prescribing antimicrobials) and in Roulette et al. (19), participants self-reported the frequency with which they used a tape measure to determine livestock weight or used a thermometer to check temperature to ensure milk is pasteurised (18, 19). Self-reporting increases the risk of bias and the likelihood that people will over-report desirable behaviours (40). The use of an objective measurement such as antimicrobial dosage charts and weight tape measures (19) can minimise such bias and increase the objectivity of the study. Where feasible, the use of objective measurements is therefore recommended in measuring outcomes of interest in interventions.

Lack of clarity in reporting was observed in two studies (18, 21). Chinwe et al. (21) reported differences in *Escherichia coli* load in chicks given a feed additive with and without antimicrobials on day 20 and 40, but information on the study setup and the administration of the antimicrobial feed additive was not provided (21). Ascertaining whether the day 20 and 40 chicks were the same chicks at two different time points or if the testing was simultaneous in two different groups was not possible. There were also some discrepancies between in text and table numbers. No statistics were performed to determine if the observed numerical differences were significant (21). In Musoke et al. (18), a two-day workshop on knowledge of AMR and AMS was conducted for medical health professionals and animal health professionals, but it was unclear whether animal health professionals were included in the assessment of feedback conducted later (18). Although this does not impact the interventions, it limits the reader’s understanding of the changes in the outcome measures of the intervention and reduces the reproducibility. Clarity in reporting interventions is vital to ensuring that knowledge gained through the intervention can be utilised to its fullest potential.

The information available can impact the documentation of an intervention. The reporting by Bangura et al. (22) was unclear due to the gaps in the reported information (22). This study examined a livestock surveillance strategy in Sierra Leone and reported clinical illnesses and the use of antimicrobials. Statistics were performed as part of the reporting. However, the statistics were based on the number of animals reported as ill and not the available general livestock population. Even though determining the denominator of the general livestock population may require additional resources, having information on the general number of the available animals can enhance the understanding of the extent of AMU in the study population.

### 3.5 Strength of evidence and assessment of bias risk

The MMAT (17) tool was used to evaluate the quality of study design of the reviewed studies (Table 6). The responses to the MMAT questions illustrated a lower quality of study design in the reviewed studies. The qualitative study by Frumence et al. (20) was the only study with affirmative responses (yes) to all questions (20). The least number of affirmative responses (yes) was recorded in one of the studies that used no comparison for before and after the intervention (18) and in the case–control study (21) suggesting a lower quality of the study designs. The lower quality of study design observed in AMR interventions in the animal health sector is also reported in AMR interventions in the human health sector in SSA. A systematic review of 28 studies appraising AMS interventions in African hospitals found the study designs to be of low quality (e.g., limited use or lack of control group, randomisation, and assessment of intervention sustainability) (41). Having a low-quality study design limits the ability to infer if any observed change in intervention outcomes is real and limits the potential to extrapolate findings.

**Table 6:** Study design quality by study.

Study	Study design	MMAT score (Question number and response)
Bangura et al. (22)	Descriptive	S.1. Yes S.2. Yes 4.1 Yes 4.2 Yes 4.3 Yes 4.4 No 4.5 Yes
Chinwe et al. (21)	CCS^a^	S.1. Yes S.2. Yes 3.1 Yes 3.2 No 3.3 Yes 3.4 No 3.5 Cannot Tell
Frumence et al. (20)	Qualitative	S.1. Yes S.2. Yes 1.1 Yes 1.2 Yes 1.3 Yes 1.4 Yes 1.5 Yes
Musoke et al. (18)	NCBA^b^	S.1. Yes S.2. Yes 3.1 Yes 3.2 No 3.3 Yes 3.4 No 3.5 Cannot Tell
Roulette et al. (19)	NCBA^b^	S.1. Yes S.2. Yes 3.1 Yes 3.2 Yes 3.3 Yes 3.4 Yes 3.5 Cannot Tell

^a^CCS = Case control study.

^b^NCBA = Non-controlled before and after.

### 3.6 Strengths and limitations of the review

A major strength of this review study was the close examination of the existing evidence on interventions aimed at tackling inappropriate AMU and the development and spread of AMR within the animal health sector in SSA. The findings provide useful insights for designing and implementing future AMR interventions in this setting. Limitations were observed in this review. A key limitation was the presence of few studies that investigated AMR interventions in the animal health sector in SSA, which suggests a need for additional but also well-designed and robust studies on the topic in SSA. The use of robust study designs may entail use of a control group, use of randomised design and having follow-up periods. Secondly, the interventions and study design of the reviewed studies were all different, which is not surprising. The variation in interventions and study designs allows for a very broad overview of ongoing work in this area but also makes comparison of studies difficult. There were also differences in the quality of the study designs, which made it difficult to compare outcomes and to assess whether the study outcomes were achieved. Thirdly, language of publication was a limitation. Articles that were not published in English language were excluded. Therefore, the authors could not establish if articles published in other languages were eligible and whether those articles would broaden the knowledge base.

## 4 Conclusion

In conclusion, only five interventions aimed at tackling inappropriate AMU and the development and spread of AMR in SSA were reported and reviewed. The interventions primarily focused on change in knowledge of AMU practices, AMR, and AMS. Other intervention outcomes included changes in uptake of AMS and use of surveillance strategies for AMU and AMR. The above findings suggest a gap in evidence of other relevant AMR intervention outcomes, such as change in AMU and change in development and/or spread of AMR. A range of motivators and barriers for the adoption of interventions were reported, including social responsibility which is defined as the desire to present interventions in a socially acceptable way, along with personal circumstances. This finding suggests that ensuring that interventions are co-created and tailored to communities and individuals’ personal circumstances may help facilitate their implementation and success. The lack of accessibility, finance, and sustainability were identified as barriers to the adoption of interventions. Concerns around the sustainability of donor-funded projects were documented, specifically concerns of funding running out or being withdrawn, which can inhibit the continuation of the intervention. Considering these potential barriers during development of future interventions may increase the chances of success of implementation of AMR interventions.

The impact of the interventions was documented only in a few studies, and no societal impact was assessed. Data on the impact of interventions is needed by decision-makers to aid decisions regarding the suitability of the interventions. Future studies of AMR interventions are therefore encouraged to consider assessing and reporting the impact (socio-economic, societal, and cultural) of AMR interventions. The observed strengths of the reviewed studies included utilising diverse study populations and the use of One Health approach which allows for consideration of a range of insights from different stakeholders and across various sectors. Multiple weaknesses were identified in some of the studies including self-reporting of outcome measures, lack of clarity in reporting, and low quality of the study designs. Future studies of AMR interventions for animal health professionals and farmers in sub-Saharan Africa should consider addressing these weaknesses in the design and implementation of the interventions.

## Data Availability

The generated dataset for this study can be provided by authors upon request.
