# Supplementary material for: Interventions for reducing antimicrobial resistance in livestock in sub Saharan Africa: systematic review
Source: Front Vet Sci. 2025 Dec 17;12:1702427. doi: 10.3389/fvets.2025.1702427 (PMC12753374; doi:10.3389/fvets.2025.1702427)
Supplement: Supplementary file 2 [file Table_2.DOCX]

Supplementary Material

**Supplementary Table 2:** Search Strategy

| Search Key | Scopus | PubMed | W of S |
| --- | --- | --- | --- |
| Antibiotic AND veterinarian AND Africa AND intervention | 2 | 12 | 2 |
| Antibiotic AND farmer AND Africa AND intervention | 3 | 0 | 8 |
| Antibiotic AND livestock AND Africa AND intervention | 5 | 2 | 22 |
| Antibiotic AND animal health professional AND Africa AND intervention | 5 | 16 | 4 |
| Africa AND intervention AND animal AND antibiotic | 45 | 1 | 61 |
| Africa AND stewardship AND animal AND antibiotic | 37 | 12 | 49 |
| Antibiotic AND paraveterinarian AND Africa AND intervention | 0 | 0 | 0 |
| Antimicrobial AND veterinarian AND Africa AND intervention | 2 | 15 | 3 |
| Antimicrobial AND farmer AND Africa AND intervention | 2 | 0 | 11 |
| Antimicrobial AND livestock AND Africa AND intervention | 5 | 4 | 24 |
| Antimicrobial AND animal health professional AND Africa AND intervention | 2 | 0 | 3 |
| Africa AND intervention AND animal AND antimicrobial | 27 | 2 | 48 |
| Africa AND stewardship AND animal AND antimicrobial | 38 | 17 | 57 |
| Antimicrobial AND paraveterinarian AND Africa AND intervention | 0 | 0 | 0 |
